# The Effect of Affective Exercise Experiences and Environmental Factors on Adherence to Outdoor Exercise Programs

**DOI:** 10.3390/ejihpe15030031

**Published:** 2025-02-28

**Authors:** Sofia Marini, Raffaele Zinno, Guido Belli, Erika Pinelli, Laura Bragonzoni, Stefania Toselli, Panteleimon Ekkekakis, Giovanni Alberto Monguzzi, Martino Belvederi Murri, Federica Folesani, Pasqualino Maietta Latessa

**Affiliations:** 1 Department of Medicine and Aging Sciences, ‘G. d’Annunzio’ University of Chieti-Pescara, 66100 Chieti, Italy; sofia.marini@unich.it; 2 Department for Life Quality Studies, University of Bologna, 47921 Rimini, Italy; erika.pinelli2@unibo.it (E.P.); laura.bragonzoni4@unibo.it (L.B.); stefania.toselli@unibo.it (S.T.); pasqualino.maietta@unibo.it (P.M.L.); 3 Department of Kinesiology, Michigan State University, East Lansing, MI 48824, USA; ekkekaki@msu.edu; 4 Department of Neuroscience and Rehabilitation, University of Ferrara, 44121 Ferrara, Italy; giovannialb.monguzzi@edu.unife.it (G.A.M.); blvmtn@unife.it (M.B.M.); federica.folesani@unife.it (F.F.)

**Keywords:** affective exercise experience, exercise adherence, physical activity, community-dwelling adults, green spaces, environmental exposure

## Abstract

Background: This study aimed to investigate the association between Affective Exercise Experiences (AEEs), environmental factors, and adherence to a structured public health exercise initiative. Methods: A prospective experimental design was employed in a primary care setting within green parks in a community in Northern Italy. Community-dwelling individuals who participated in a 15-week outdoor exercise program were recruited. Participants engaged in 43 low-to moderate-intensity outdoor exercise sessions, held three times a week from May to July 2022, with each session lasting 60 min. A total of 68 participants (mean age: 64.6 ± 8.6 years) completed the questionnaires. Results: A causal analysis revealed a small but significant positive effect of AEEs on adherence, with higher AEE scores associated with an increased likelihood of attending exercise sessions. The predictive model identified a combination of AEE scores, environmental temperature, distance from the exercise site, and weekday as significant predictors of adherence. Conclusions: This study highlights the importance of AEEs, as well as environmental and demographic factors, in predicting adherence to structured exercise programs. The findings suggest that fostering positive affective experiences related to exercise could enhance adherence, particularly among older adults.

## 1. Introduction

Physical activity (PA) and exercise behavior are key lifestyle factors with significant implications for health prevention and promotion, including the reduction in disease burden. Individuals embracing an active lifestyle have several benefits, including enhanced physical and mental well-being (I. M. Lee et al., 2012; McTiernan et al., 2019; Schuch et al., 2016). Despite these benefits, performing regular PA and exercise remains a challenge for the majority of the individuals, who often lead sedentary lives (H. H. Lee et al., 2016). Understanding the factors that influence adherence to structured exercise programs is crucial to promote active lifestyles. The initiation and maintenance of PA or exercise behavior involve a complex interplay of individual-related, socioeconomic, exercise-related, and environmental factors (Calonge Pascual et al., 2022; Junge et al., 2016; Pavey et al., 2012; Ricke et al., 2023). These factors can be broadly categorized as intrinsic, related to participants’ lifestyle, physical or psychological characteristics (e.g., physical health, motivation, preferences), and extrinsic, concerning elements characterizing the personal or shared environment (e.g., family or work demands, living conditions, exercise setting). Regular participation in a physical activity program is vitally important for the population also to prevent decline in mobility function (Hicks et al., 2012). However, adherence to an exercise program is problematic in all age groups, particularly among older adults. For instance, a meta-analysis of 127 exercise interventions for older adults demonstrated that, within the first three to six months, 40–65% of the participants will drop out (Dishman & Buckworth, 1996).

Recent systematic reviews suggest that people, especially those with mental or physical disorder, can obtain health benefits if they use and are exposed to natural outdoor environments (Coventry et al., 2021). Therefore, access to green spaces provides opportunities to support and promote public health and well-being (Grigoletto et al., 2021). The promotion of regular outdoor exercise programs rather than indoor ones may be a more effective approach to increasing PA levels, particularly given that few individuals are meeting PA guidelines (Thompson Coon et al., 2011). In particular, the outdoor natural green environment can help people to increase their adherence to exercise programs (Marini et al., 2022), promoting social interaction and contact with nature, thereby providing multiple health benefits. In addition, environmental factors, such as air pollution, temperature, and climatic conditions also influence the propensity of an individual toward performing outdoor activity (An et al., 2018). Indeed, temperature differences, precipitation, and sunlight exposure have been reported as factors influencing the amount of PA and sedentary behavior (Park et al., 2021).

Participation in exercise programs also depends on psychological factors, both in the “cognitive” and “affective” domains. Recent theories on the construct Affective Exercise Experiences (AEEs) (Ekkekakis et al., 2020) underscore the significance of incorporating “affect” into the assessment of factors influencing the initiation, experience, response to PA and exercise behaviors (Hong et al., 2018; Lachowycz & Jones, 2013; Tamosiunas et al., 2014). AEEs refer to the overall emotional responses, ranging from pleasant to unpleasant, that a person associates with exercise throughout their life (Ekkekakis et al., 2021). This concept captures how previous experiences and the associated emotions influence an individual’s current perception and feelings toward exercise. The AEEs can be assessed by the Affective Exercise Experiences (AFFEXX) questionnaire. The AFFEXX is based on the hypothesis that core AEEs (pleasure–displeasure, energy–tiredness, calmness–tension) are influenced by six antecedent appraisals and, in turn, shape attraction or antipathy towards exercise (Ekkekakis et al., 2021). Specific affective dimensions such as emotions, mood, and core affect, are increasingly recognized as relevant to describing, predicting or explaining exercise adherence, announcing a new era of “affectivism” (Dukes et al., 2021). However, there is still a lack of knowledge about the relationship between the AEEs and the adherence to structured exercise sessions, particularly in relation to the older population, outdoor activities, and environmental factors.

In this context, it is crucial to estimate the extent to which AEEs influence behavior, i.e., the strength of its causal association (Pearl, 2009). Additionally, empirically estimating the joint predictive value of AEEs and other environmental variables, i.e., their accuracy in predicting exercise adherence in the real world (Teixeira et al., 2023). The objective of this study was to investigate the association between the AEEs, environmental factors, and adherence to a structured public health exercise initiative.

The primary aim was to examine the potential causal relationship between AEEs and adherence to exercise. The secondary aim was to examine the predictive value of AEEs by developing a predictive model for adherence that leverages information on both individual factors (i.e., distance from the site, psychological factors, perception, attitudes, and motivation), and one environmental factor, i.e., environmental temperature.

We hypothesized that (a) baseline self-reported AEEs would be positively associated with adherence to exercise, namely, with a higher number of attended sessions assessed prospectively; and (b) using information on AEEs and other individual and environmental factors would allow us to predict future attendance to exercise at the individual level.

## 2. Materials and Methods

### 2.1. Research Design and Participants

A prospective experimental study was conducted to investigate the association between the AEEs and adherence to a structured public health exercise initiative. The study sample was composed of community-dwelling individuals who participated in structured exercise programs in an open and green environment from May 2022 to July 2022. All the activities were performed in green parks placed in Vignola (Italy). The project was promoted by the Local Health Union Company of Modena (Italy), the Sports Medicine and the District of Vignola, the “Terre di Castelli Union” and the Municipality of Vignola, which provided advertisements and community outreach in order to recruit the population. This study established the following eligibility criteria:

Inclusion Criteria:

- Adult population aged >18 years old having the willingness to participate in the exercise sessions.

Exclusion Criteria:

- Any contraindications to the participation in a low to moderate-intensity physical activity program.
- Any alterations in communication skills and/or sensory functions so severe as to make it impossible to understand and/or execute the instructions given by the trainer (dementia, aphasia, blindness, deafness).

Participants meeting the inclusion criteria signed an informed consent form, and this study was executed in accordance with the Helsinki Declaration. The University of Bologna Bioethics Committee granted approval for this study (Prot. No. 0169182). Patients or the public were not involved in the design, conduct, reporting, or dissemination plans of our research.

### 2.2. Exercise Program

The exercise program was centered around a series of freely accessible activities held at fixed and well-distributed times, from Monday to Saturday in 2022, during the spring/summer period, in various parks and gardens in the municipal area. Specialized trainers (Certified Kinesiologist) in exercise science oversaw and monitored the exercise sessions. Their presence was crucial in determining the appropriate dosage of exercise tailored to the target group, considering individual variations in physical efficiency. The proposed activities did not involve intricate movements or complex motor tasks, and both the exercise intensity and workload volume were generally kept at low-to-moderate levels. In order to ensure consistency among kinesiologists, brief training has been provided, aimed at sharing the same goals and the same strategies to reach them. The type of exercises proposed included a wide range according to each phase of the session such as the following for the warm-up: cardio-respiratory exercise conditioning, multi-articular exercises able to safely solicit all the main muscle groups increasing body temperature and metabolism, joint mobilization, upper and lower limb coordination, proprioception, balance and postural education; the following for the central phase: aerobic activity within the walking group; and, finally, the following for the cool-down and stretching: predominantly exercises in an upright position, able to stretch the main muscles, and holding a stretch position for up to 30 s.

The entire exercise program lasted 15 weeks, with 3 exercise sessions per week, totaling 43 scheduled outdoor exercise sessions.

### 2.3. Research Tools

Participants completed a motivational questionnaire based on the framework from a previous study (Ekkekakis et al., 2021) to identify perceptions, attitudes, intrinsic and extrinsic factors related to the environment that participants recognized as enhancing their motivation to exercise in nature. Furthermore, participants filled out the AFFEXX to assess different components of motivation across different modes of exercise programs in green environments. The questionnaires were administered through an ad hoc internet survey and email response. Participants were also asked to rate items on their motivations to practice exercise, perceptions, and attitudes towards exercise programs in green environments. The questionnaire was administered once, before the beginning of the exercise program.

#### 2.3.1. Adherence to Structured Exercise Sessions

The overall adherence to exercise sessions was defined as the recorded number of attended sessions out of the total number of scheduled sessions. The presence or absence of the participant was recorded by the trainers during each session.

#### 2.3.2. Collection of Temperature Data

This assessment instrument was developed within the theoretical framework of the Affective-Reflective Theory of physical inactivity and exercise (ART) (Brand & Ekkekakis, 2018; Ekkekakis et al., 2021), which assumes that exercise experiences have permeated the stimulus concept of “exercise” with pleasant or unpleasant valence.

The AFFEXX was composed of 36 items (Ekkekakis et al., 2021), each scored on a scale ranging from 1 to 7. It includes 10 subscales, with particular emphasis on the Attraction/Antipathy Subscale (AAS). The AAS may result from the interaction of various cognitive and affective appraisals and is specifically involved in the final decision to pursue PA and exercise. Therefore, we used the AAS as a proxy measure of AEE.

#### 2.3.3. Assessment of Affective Exercise Experience

The data on daily temperatures from April to July, the period of the PA sessions, were collected from the Arpae Emilia-Romagna website (https://www.arpae.it/it/dati-e-report/richieste-e-forniture-dati Accessed on 27 January 2023).

### 2.4. Statistical Analysis

We addressed data analyses with an explanatory and a predictive approach.

For the primary aim, we assessed the potential causal effect of AEEs on adherence, adopting the causal inference approach and analytic tools (Cunningham, 2021). We developed a simple Directed Acyclic Graph (DAG) to transparently represent the assumptions on causal relationships between variables, including AEEs and exercise adherence. In the causal inference framework, the analysis of a DAG facilitates identifying variables as confounders, mediators, or colliders. This supports the choice of adjustment variables in the subsequent analyses that are used to estimate the causal effect and reduces the risk of bias (Cinelli et al., 2020). To this end, we employed the 0.2.1 ggdag R package that provides the minimal adjustment set for a given DAG (Barrett, 2018). To obtain an estimate of the total causal effect, analyses need to be adjusted for confounders but not mediators or colliders. The AAS was used as the measure of AEEs (exposure), while the presence/absence of each individual in the exercise sessions was used as the measure of adherence (outcome).

We estimated the strength of the causal relationship between AEEs and adherence using a Bayesian Generalized Multilevel Linear Model with Bernoulli outcome distribution. All Bayesian models were fitted with the 2.19.0 version of the brms R package using default uninformative priors (Bürkner, 2017). The attendance to each session (yes/no) was used as the outcome. Attendance to multiple sessions was nested within individuals, which served as a varying (random) effect. The mixed model approach allows us to regularize parameter estimates and to reduce the impact of individual unmeasured confounders, as well as to account for the uncertainty of each parameter of the model (McElreath, 2018). As a result, the strength of the relationship between AEEs and adherence across subjects (the fixed effect) was estimated more reliably. We reported this estimate as the marginal effect of AEEs on adherence, i.e., the probability of attendance to each session of exercise for a unit increase in the AAS, holding all other variables in the model constant at the median (Arel-Bundock, 2023). Also, we report data on the posterior distribution, i.e., 80% and 95% credibility intervals.

Second, we developed a preliminary predictive model of exercise adherence. Here, the focus was not on reducing bias in the estimation of a specific effect but rather on maximizing the available information from variables to predict the adherence levels optimally while reducing the risk of overfitting. We fitted a series of Bayesian Generalized Multilevel Linear Models, again based on a Bernoulli outcome distribution. The initial model consisted of a simple intercept where the mean population attendance is fixed, varying only across individuals as a random factor. Then, subsequent models were constructed by adding predictors and assessing whether model predictive accuracy improved. The models’ predictive accuracy was compared using Pareto-Smoothed Importance Sampling (PSIS) Leave-One-Out Cross-Validation (LOO-CV). This approach not only assesses the model fit to the dataset but also identifies the best-fitting model in terms of predictive performance on unseen data, mitigating the risk of selecting overfitting models (Vehtari et al., 2017). In particular, we added the following: (i) weekday as an additional random factor (cross-classified model); (ii) interaction terms between other variables as fixed factors (e.g., between temperature and AEEs); (iii) the distance from exercise site, entered as a monotonic effect; and (iv) random slopes of the effects of AEEs and day temperature (effects that vary between subjects or between weekdays). We report the final model’s accuracy by comparing the median posterior probability of attendance at each session, with the actual attendance. We used the receiver operating characteristic (ROC) curve analysis to examine the most accurate threshold of probability to predict attendance.

## 3. Results

### 3.1. Demographic Characteristics

The people involved in exercise sessions were asked to participate in this study, and only those who expressed the willingness to participate were included in this study. Among 80 persons, 12 refused to participate; thus, a total of 68 participants (mean age = 64.6 ± 8.6) completed the questionnaires and were included in the analysis. No missing data were detected. The analysis of the sample characteristics did not show relations between gender and other parameters, such as depression, smoke, alcohol, distance from home, and comorbidities. In addition, the results of the Attendance, Attraction, Pleasure sub-score of the AFFEXX were reported divided by gender in Table 1.

### 3.2. Causal Effect of AAS on Adherence

**Figure 1:**
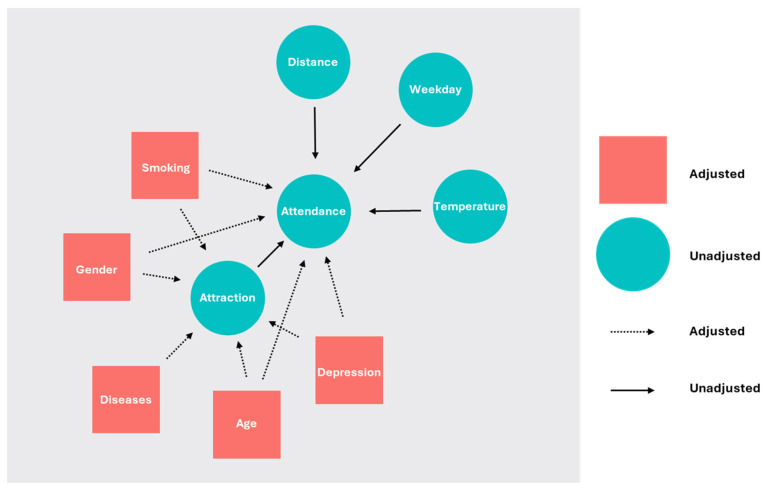
Directed acyclic graph depicting the causal assumptions of exercise attitude on attendance.

The model estimating the causal effect of AAS on adherence converged well (Supplementary Materials Table S1). As suggested by the DAG analysis, it was adjusted by age, gender, smoking, disease status, and depression (Figure 1). The effect of AAS (Median = 0.18, 95% CI [−0.01, 0.38]) had a 96.92% probability of being positive (>0) and 83.01% of being significant (>0.09). The median standardized effect was overall small (0.18, 95%CrI: −0.01–0.38). In the response scale, an increase from 2 to 7 in the AAS would correspond to an increase from an 11% (4–24%) to 24% (13–38%) probability of attending one exercise session, that is, a mean increase from 1.1 to 2.4 session over ten sessions.

### 3.3. Predictive Model of Adherence

**Figure 2:**
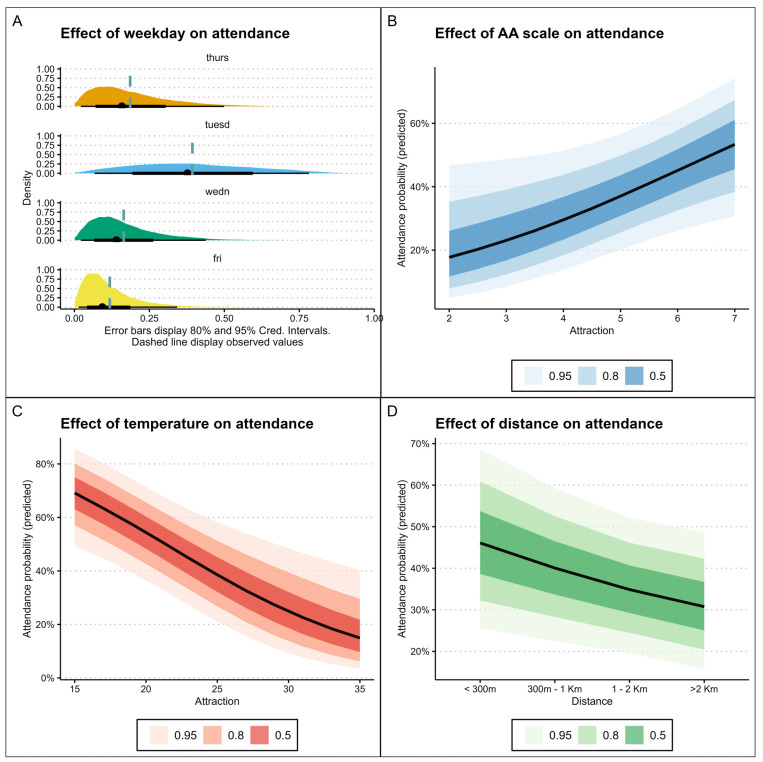
Marginal effect of AAS on probability of attending each session: (**A**) effect of weekday on attendance; (**B**) effect of AA scale on attendance; (**C**) effect of temperature on attendance; and (**D**) effect of distance on attendance.

We developed a series of models to predict adherence for each exercise session, with detailed model specifications provided in Supplementary Materials (Table S2). Briefly, the model demonstrating the best out-of-sample predictive performance nested attendance within subjects and weekdays. This model incorporated fixed effect predictors such as the AAS score, AFFEXX pleasure subscale, day temperature, distance from exercise site, age, gender, depression, cigarette smoke and presence/absence of physical diseases. Additionally, the model included varying random slopes of temperature across weekdays and subjects (model summary in Supplementary Materials Table S3). The score in the AAS, distance, and outdoor temperature from the site were significantly associated with the probability of attending (Figure 2). Concerning environmental factors, air humidity was also considered, but it was highly correlated with temperature and did not add information or a predictive value to the models.

Also, there was considerable variation across individuals according to the day of the week. Interestingly, the predictive value of the AAS score on the probability of attendance was highest at lower temperatures and decreased at the higher temperatures (Supplementary Materials Figure S1).

Through optimization, a probability of 24.3% was identified as the cut-off point for the model. In other words, when the model predicts a probability of attendance of 24.3% or higher, it should be interpreted as indicative of attendance. At this cut-off, the model demonstrated adequate accuracy (accuracy: 74.6%, AUC ROC: 0.782; sensitivity: 66.2%; specificity: 76.9%) and is adequately calibrated for lower and higher values of attendance (Supplementary Materials Figures S2 and S3).

### 3.4. Adherence Levels

**Figure 3:**
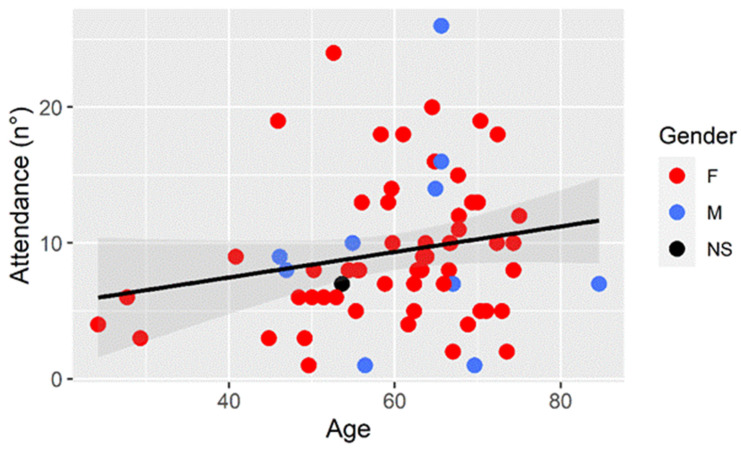
Relation among attendance, age, and gender of the participants.

Participants attended exercise sessions on average 9.2 ± 5.5 times with no significant differences noted between males and females. While a weak positive correlation (r = 0.195) with participant age was observed, it did not reach statistical significance (*p* > 0.05) (Figure 3).

The presence of comorbidities (Supplementary Materials Figure S4) showed no significant impact on attendance (*p* > 0.05). Similarly, there were no significant differences in attendance between participants who drank alcohol (mean of 9.0 ± 4.2) and those who did not (mean of 9.4 ± 6.4) (Supplementary Materials Figure S5). Notably, a shorter distance from home to the park was associated with higher attendance (Supplementary Materials Figure S6).

**Figure 4:**
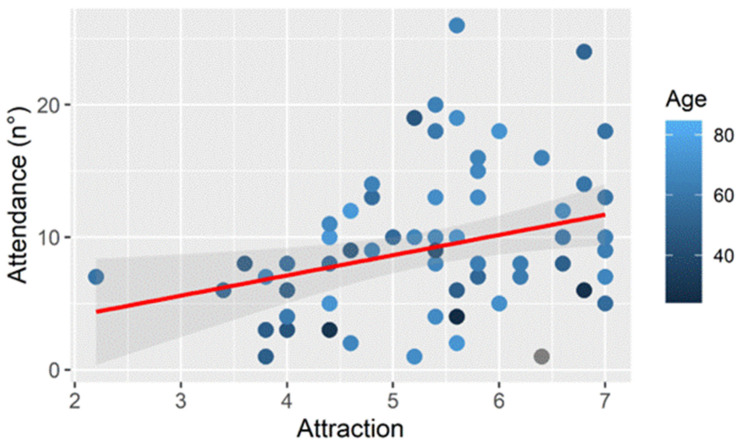
Relationship between attendance to sessions and Attraction sub-score.

Examining the relationship between attendance and AFFEXX (Attendance–Attraction), it was found that participants with a higher Attraction sub-score attended more exercise sessions (r = 0.301, *p* < 0.05) (Figure 4).

No significant gender-based differences were observed in this context (Table 1). All correlations between the AFFEXX sub-scores and attendance are thoroughly presented in the Supplementary Materials (Figure S7). Notably, the pleasure sub-score did not show a significant correlation with attendance (r = 0.204, *p* > 0.05) (Figure S9).

Furthermore, a compelling association emerged between attendance, environmental temperature, and weekday. Participants exhibited higher adherence when the temperature ranged between 25 and 30 degrees (Supplementary Materials Figure S8). Regarding weekdays, Tuesday recorded the highest turnout, while Friday had the least attendance (Supplementary Materials Figure S9).

## 4. Discussion

This study investigated the association between the AEEs, environmental factors, and adherence to a structured public health exercise initiative. Regular outdoor exercise can be beneficial for several reasons (Guarda-Saavedra et al., 2022; Wang & Li, 2024). However, participation in outdoor exercise can be affected by a number of factors that are difficult or impossible for an individual to control, including built environment (Humpel et al., 2002; Moschny et al., 2011; Tucker & Gilliland, 2007; Witham et al., 2014), day length (Witham et al., 2014), and weather patterns such as temperature, wind intensity, and precipitation (Brocherie et al., 2015; Chan & Ryan, 2009). Indeed, weather circumstances emerge as significant predictors of PA, with increased daily temperatures, longer day lengths, and favorable weather associated with increased PA, particularly in older individuals, as evidenced by accelerometer research (Klimek et al., 2022). Conversely, unpleasant weather tends to decrease PA levels, especially in less trained older adults compared to their fitter counterparts. Additional factors such as low sunshine duration, extreme daily temperatures, and varying levels of rainfall have been linked to lower PA levels (Klimek et al., 2022).

Previous studies revealed that barriers to exercise adherence include the inconvenient timing of sessions, their cost, and location beyond the weather conditions (Hicks et al., 2012; Wagner et al., 2019).

These factors, which may interact with each other, are postulated to affect PA levels by influencing an individual’s perception of the safety of being outdoors, the comfort in exercising, and the suitability of the neighborhood environment to exercising (Wagner et al., 2019).

In this context, the present study reveals that AEEs seem to hold both a casual predictive value on adherence to exercise, together with other social and environmental factors. In particular, when exercise attraction is combined with distance from the exercise site, weather, and weekday, it allows us to gain predictive insights into the future level of attendance to exercise sessions. These findings expand previous results and improve the knowledge on exercise programs adherence (Morgan et al., 2016).

The strength of the current study lies in the use of a novel statistical analysis and the role of self-reported AEEs alongside established environmental and demographic factors. In our opinion, this study is further strengthened by a rigorous methodological approach and use of a real-world sample of older people in exercise activities. To our knowledge, there are few examples of adopting the causal inference approach in this field of research, together with use of robust Bayesian analyses. This approach may protect from different sources of bias and promote a transparent, nuanced understanding of the complex dynamics at play.

However, it is crucial to acknowledge the study limitations, including the relatively small sample size of individuals likely to participate in similar exercise sessions. Thus, our findings may not generalize to younger, sedentary individuals or males. In addition, the small sample size prevented us from conducting predictive analyses on training and testing subsamples. Bayesian out-of-sample cross-validation might mitigate the risk of overfitting, but it does not eliminate it. Furthermore, another potential for selection bias was related to the self-reported willingness to participate and the use of self-reported measures, which might introduce response bias. Lastly, we did not assess body mass index (BMI) and depression severity, as recommended by (Rodrigues et al., 2018), which might have further improved the predictive accuracy of the model.

## 5. Conclusions

Affective responses to exercise might contribute to cause adherence, even after adjusting for potential confounding factors. Exercise attraction, distance from exercise site, weather, and weekday allow us to gain predictive insights into the future level of attendance to exercise sessions

In this framework, such studies aimed at increasing the reputation of outdoor group exercise can undoubtedly become a means for improving health and well-being and reducing sedentary behavior. Therefore, the evidence of such health benefits could become of high relevance for healthcare professionals, urban planners, and policymakers.

Future research should explore these relationships in larger and more diverse populations and consider additional factors such as BMI and depression severity to improve predictive accuracy. Furthermore, longitudinal studies to assess long-term adherence, intervention trials to test strategies for improving AEEs, and the inclusion of other environmental and psychosocial variables, such as community support or weather variability, are highly recommended.

## Figures and Tables

**Table 1:** Characteristics of the sample.

Characteristics	Total	Females	Males
	n° (%)	n° (%)	n° (%)
Sample	68 (100%)	58 (85%)	10 (15%)
Depression			
Yes	56 (82%)	10 (18%)	2 (20%)
No	12 (18%)	47 (82%)	8 (80%)
Smoke			
Yes	4 (5.9%)	3 (5%)	1 (10%)
No	64 (94%)	54 (95%)	9 (90%)
Chronic illnesses			
Yes	40 (59%)	22 (39%)	5 (50%)
No	28 (41%)	35 (61%)	5 (50%)
Alcohol			
Yes	37 (55%)	32 (56%)	5 (50%)
No	30 (45%)	25 (44%)	5 (50%)
Distance from home			
<300 m	6 (9%)	4 (7%)	2 (20%)
300 m–1 km	27 (40%)	23 (41%)	4 (40%)
1–2 km	13 (19%)	11 (19%)	1 (10%)
>2 km	22 (32%)	19 (33%)	3 (30%)
	Median (IQR)	Median (IQR)	Median (IQR)
Age	63.0 (54.0, 68.0)	62.5 (54.0, 67.7)	65.2 (55.3, 66.7)
Attendance	8.0 (6.0, 12.0)	8.0 (5.0, 12.0)	8.5 (7.0, 13)
Attraction	5.5 (4.6, 6.2)	5.6 (4.4, 6.4)	5.1 (4.7, 5.8)
Pleasure	6.3 (5.7, 7.0)	6.6 (5.5, 7.0)	6.3 (6.0, 7.0)

## Data Availability

The dataset generated during this study is available upon request.

## Supplementary Materials

The following supporting information can be downloaded at https://www.mdpi.com/article/10.3390/ejihpe15030031/s1, Table S1. Causal model of AAS on adherence; Table S2. Predictive models of adherence; Table S3. Parameters of the model predicting adherence; Figure S1. Interaction between temperature and attraction; Figure S2. Calibration plot of the predictive model; Figure S3. ROC curve plot of the predictive model; Figure S4. Relation between attendance and pathologies; Figure S5. Relation between attendance and alcohol; Figure S6. Relation between attendance and distance from the exercise site; Figure S7. Relation between attendance and all the subscales of AFFEXX; Figure S8. Relationship between attendance, weekday and temperature; and Figure S9. Percentage of session attendance per weekday.
