# Supplementary material for: The Effect of Affective Exercise Experiences and Environmental Factors on Adherence to Outdoor Exercise Programs
Source: Eur J Investig Health Psychol Educ. 2025 Feb 28;15(3):31. doi: 10.3390/ejihpe15030031 (PMC11940904; doi:10.3390/ejihpe15030031)

Table S1. Causal model of AAS on adherance

Parameter	Median	CI	CI_low	CI_high	pd	Rhat	ESS
**(Intercept)**	-3.130	0.950	-4.585	-1.709	1.000	1.001	6246.729
**Attraction**	0.184	0.950	-0.010	0.378	0.969	1.000	5647.263
**Depressione (yes)**	-0.117	0.950	-0.607	0.364	0.687	1.001	6427.819
**Age**	0.013	0.950	-0.004	0.030	0.934	1.001	6532.647
**Genere (Male)**	0.079	0.950	-0.456	0.611	0.620	1.000	6434.142
**Smoke (yes)**	-0.727	0.950	-1.734	0.258	0.927	1.000	7127.615
**Random effects**							
**Sd (Intercept)**	0.650	0.090	0.500	0.840	1.000	5917.000	9483.000

Table S2. Predictive models of adherence

Model denomination	Brms formula	ELPD Loo comparison	Bayesian R squared
**M0 multilevel random intercept model (nested within subject)**	value ~ 1 + (1|id)	-119.0 ± 15.9	7.27 (5.34 – 9.33)
**M1 added fixed predictor(s) (nested within subject)**	value ~ temperature + (1|id)	-112.2 ± 15.2	7.85 (5.80 – 10.00)
**M3 added fixed predictor(s), added nesting within weekday (cross-classified model)**	value ~ temperature + attraction + (1|id) + (1|day)	-8.4 ± 3.7	15.64 (13.10 – 18.17)
**M3.1 added interaction between temperature and attraction**	value ~ temperature + attraction + temperature: attraction + (1|id) + (1|day)	-8.8 ± 3.8	15.72 (13.13 – 18.25)
**M3.4 added the monotonic effect of distance**	value ~ temperature + attraction + mo(distance) + (1|id) + (1|day)	-8.2 ± 3.6	15.71 (13.14 – 18.22)
**M6 added fixed predictors and random slope of attraction (effect of attraction varying across subjects)**	value ~ temperature + attraction + mo(distance) + depressione + fumo + patologie + (1 + attraction | id) + (1 | day)	-9.0 ± 3.6	15.91 (13.40 – 18.44)
**M7 added random slope of temperature (effect of temperature varying across weekdays)**	value ~ temperature + attraction + pleasure + mo(distance) + depressione + fumo + patologie + (1 | id) + (1 + temperature | day)	-3.9 ± 2.2	16.42 (13.88 – 18.99)
**M8 maximal number of fixed predictors and both random slopes of temperature**	value ~ temperature + attraction + pleasure + mo(distance) + age + gender + depression + smoking + physical diseases + (1 + temperature | id) + (1 + temperature | day)	0.0 ± 0.0	17.02 (14.47 - 19.57)

Table S3. Parameters of the model predicting adherence

	Estimate	Est.Error	95%CrI lower	95%CrI higher	Rhat	Bulk_ESS	Tail_ESS
**Group-Level Effects:**							
**~day (Number of levels: 4)**							
**sd(Intercept)**	2.480	1.200	0.860	5.460	1.00	4991	6253
sd(Temperature)	0.100	0.080	0.010	0.300	1.00	3563	3866
cor(Intercept,Temperature)	-0.580	0.390	-0.980	0.460	1.00	7495	8853
**~id (Number of levels: 67)**							
sd(Intercept)	0.730	0.560	0.030	2.080	1.00	2150	6715
sd(Temperature)	0.050	0.020	0.020	0.100	1.01	629	952
cor(Intercept,Temperature)	-0.590	0.490	-0.990	0.770	1.01	480	947
**Population-Level Effects:**							
Intercept	-0.630	1.710	-3.950	2.770	1.00	7704	9273
Age	0.010	0.010	-0.010	0.030	1.00	10652	10894
Gender (Male)	-0.060	0.310	-0.670	0.530	1.00	9059	11197
**Temperature**	**-0.090**	**0.060**	**-0.200**	**0.030**	**1.00**	**5062**	**5052**
**Attraction/antipathy subscale**	**0.330**	**0.170**	**0.000**	**0.670**	**1.00**	**7452**	**10913**
Pleasure subscale	-0.090	0.190	-0.460	0.270	1.00	8685	10933
Depression	-0.090	0.280	-0.640	0.450	1.00	10827	11655
Smoke	-0.540	0.560	-1.640	0.570	1.00	10074	11569
Diseases	0.020	0.240	-0.440	0.480	1.00	9912	10764
**Distance (monotonic)**	**-0.220**	**0.110**	**-0.470**	**-0.010**	**1.00**	**8609**	**10334**
**Simplex Parameters:**							
modistance1[1]	0.360	0.210	0.020	0.800	1.00	13725	9909
modistance1[2]	0.350	0.220	0.020	0.830	1.00	16439	11383
modistance1[3]	0.290	0.210	0.010	0.770	1.00	15934	11063

Figure S1. Interaction between temperature and attraction

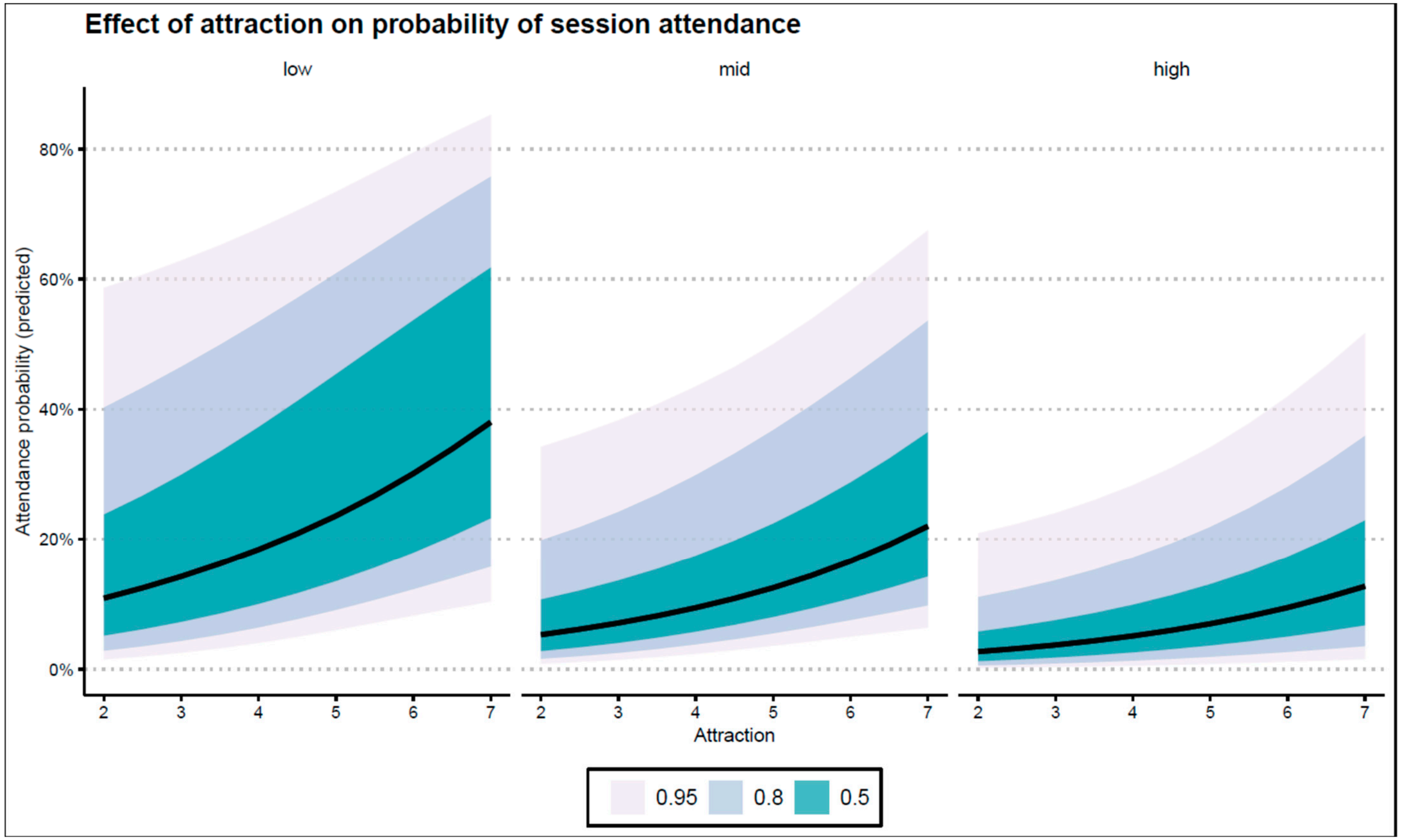

Note: Panels represent the relationship between AAS score and probability of attendance within tertiles of temperature. Y axis: posterior predictive probability of attendance. X axis: Attraction/Antipathy subscale score

Figure S2. Calibration plot of the predictive model

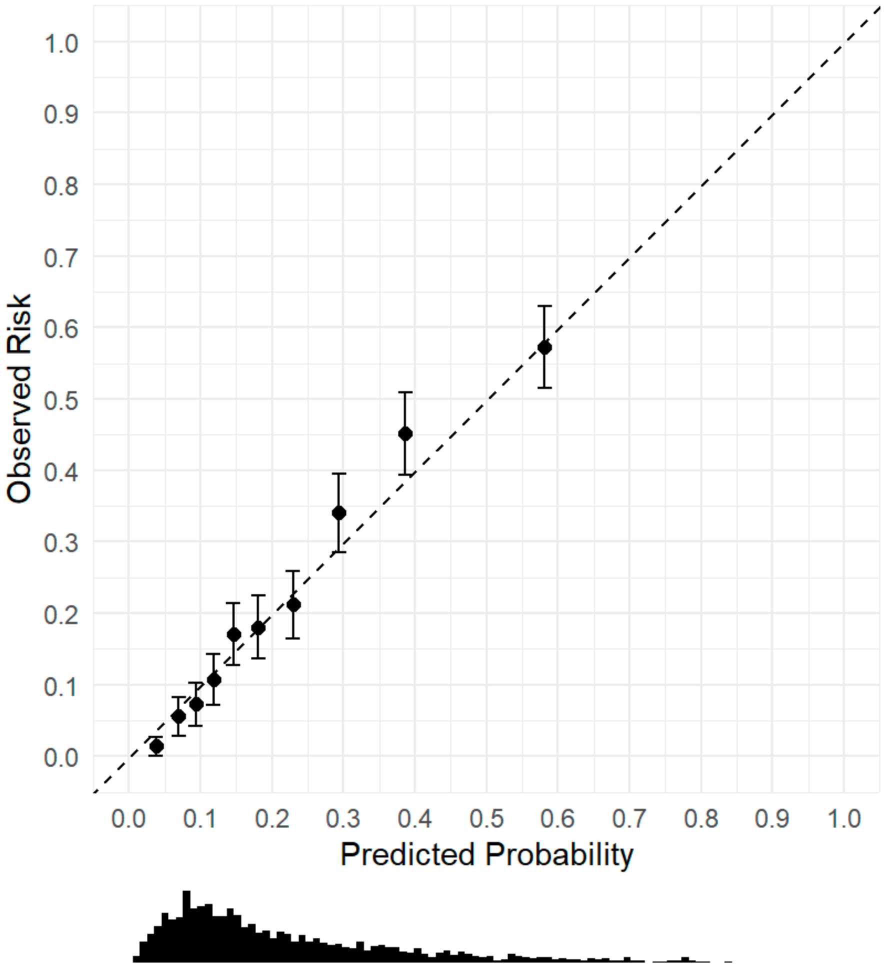

Figure S3. ROC curve plot of the predictive model

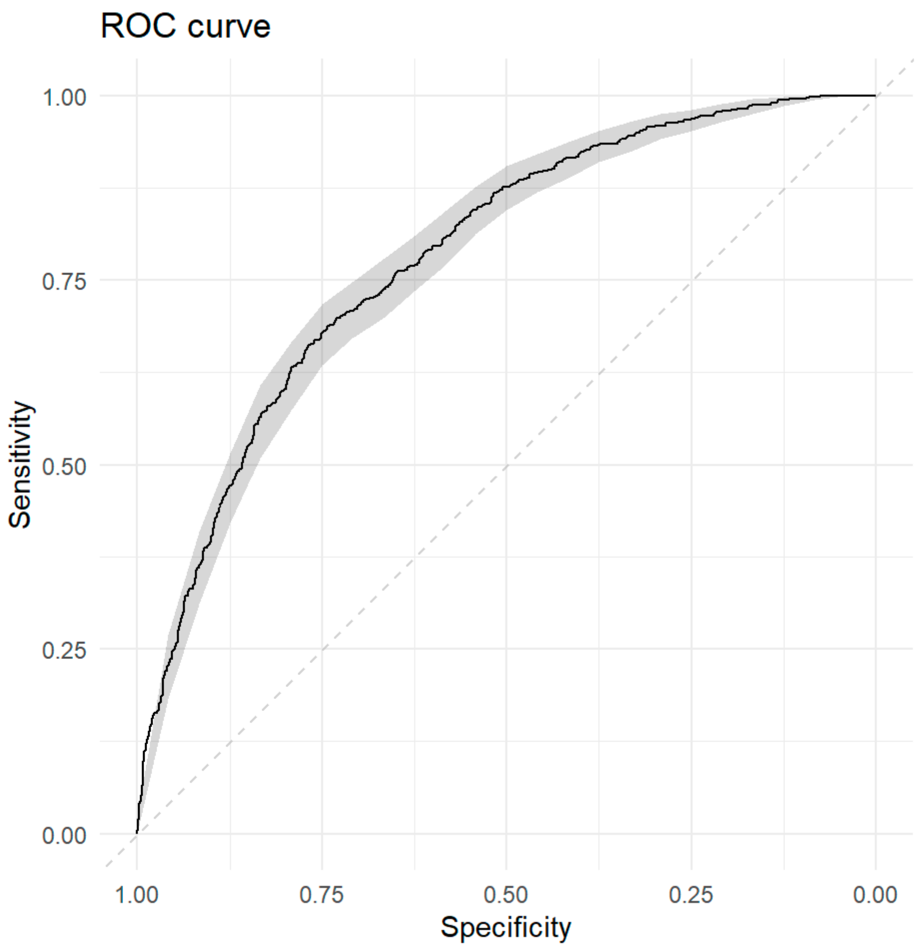

Figure S4. Relation between attendance and pathologies

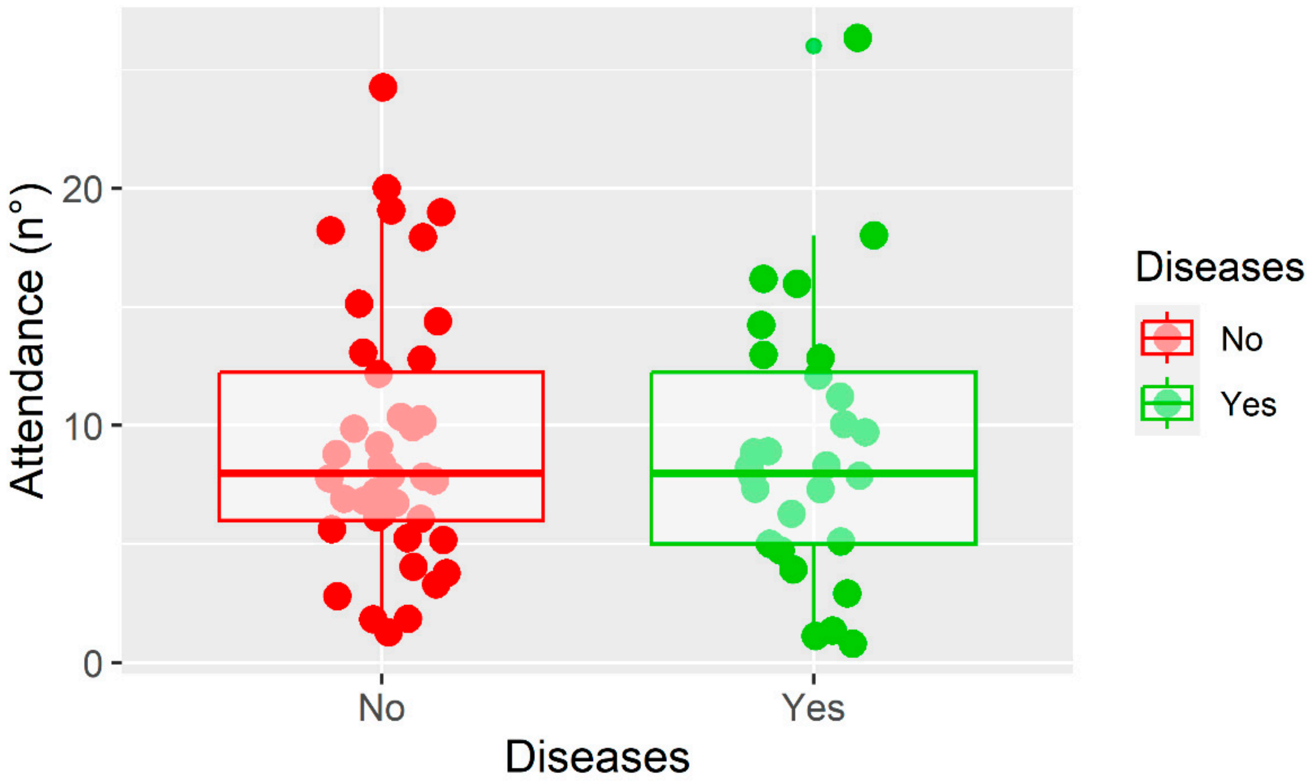

Figure S5. Relation between attendance and alcohol

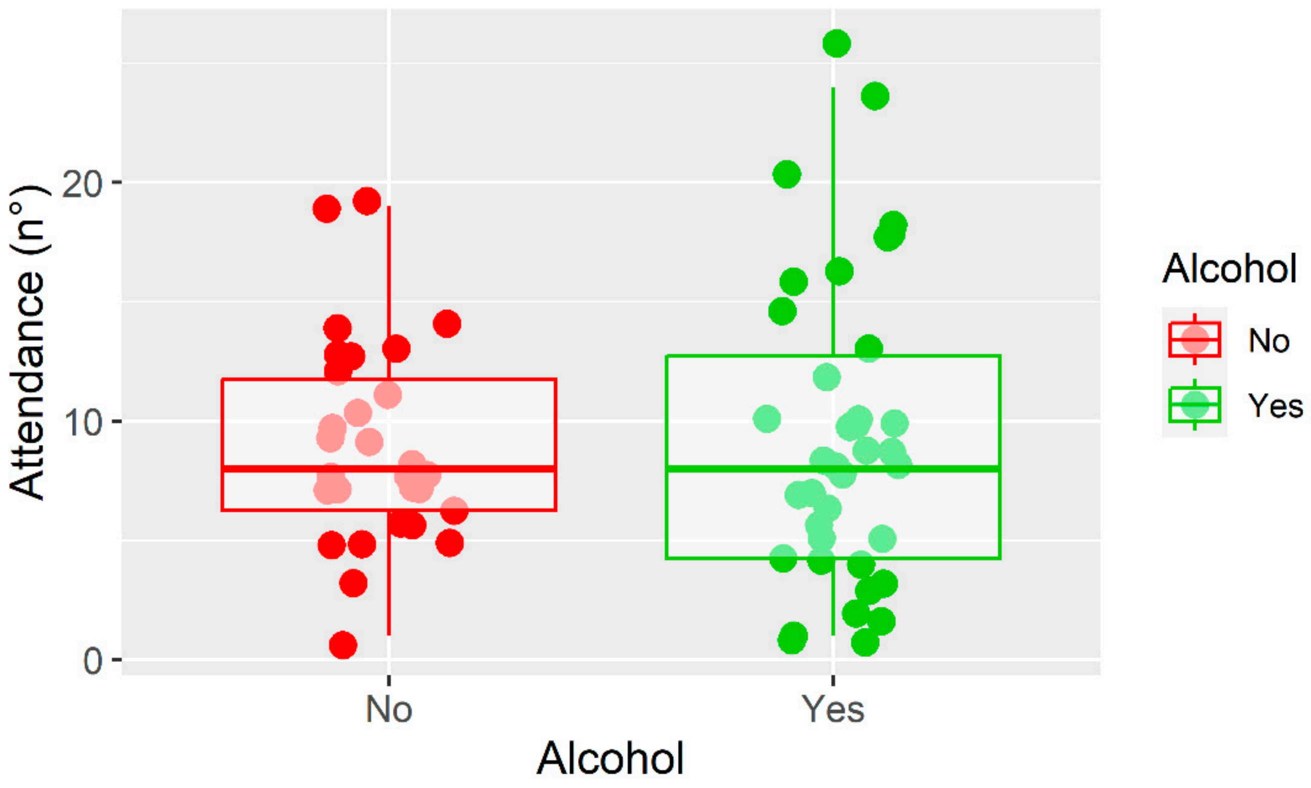

Figure S6. Relation between attendance and distance from the exercise site

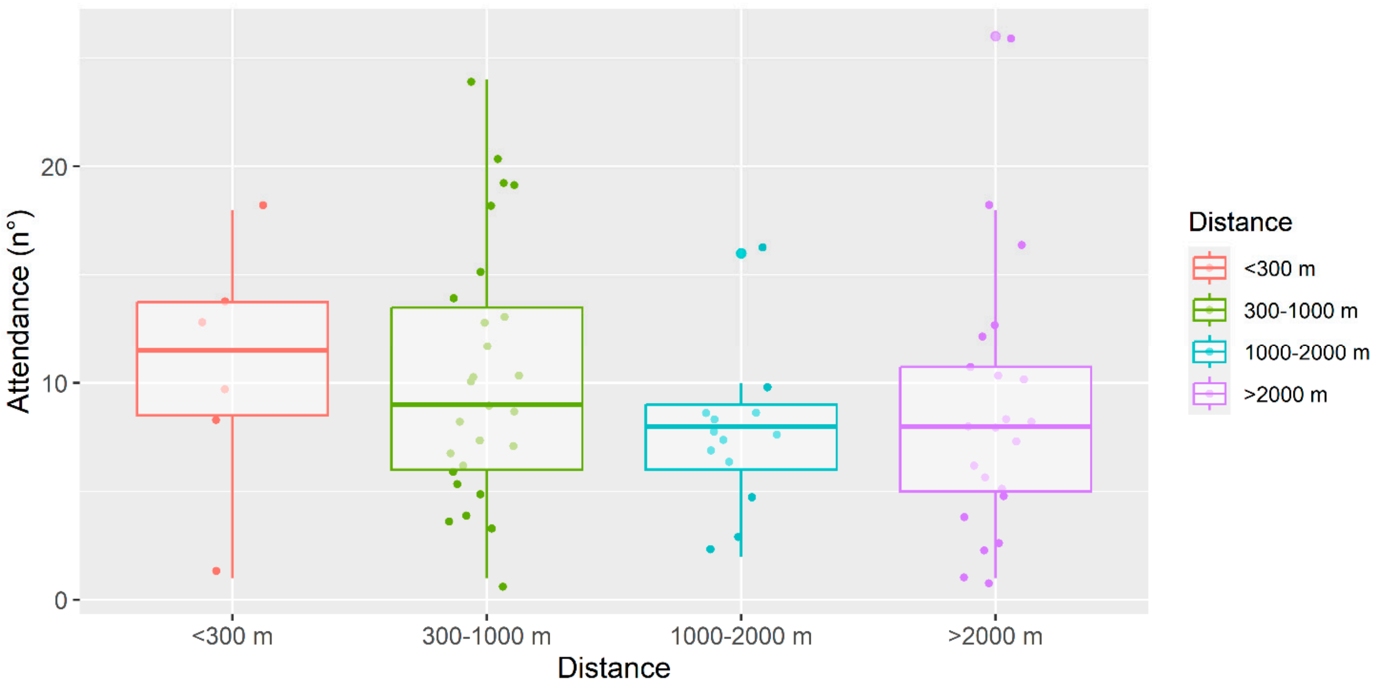

Figure S7. Relation between attendance and all the subscales of AFFEXX.

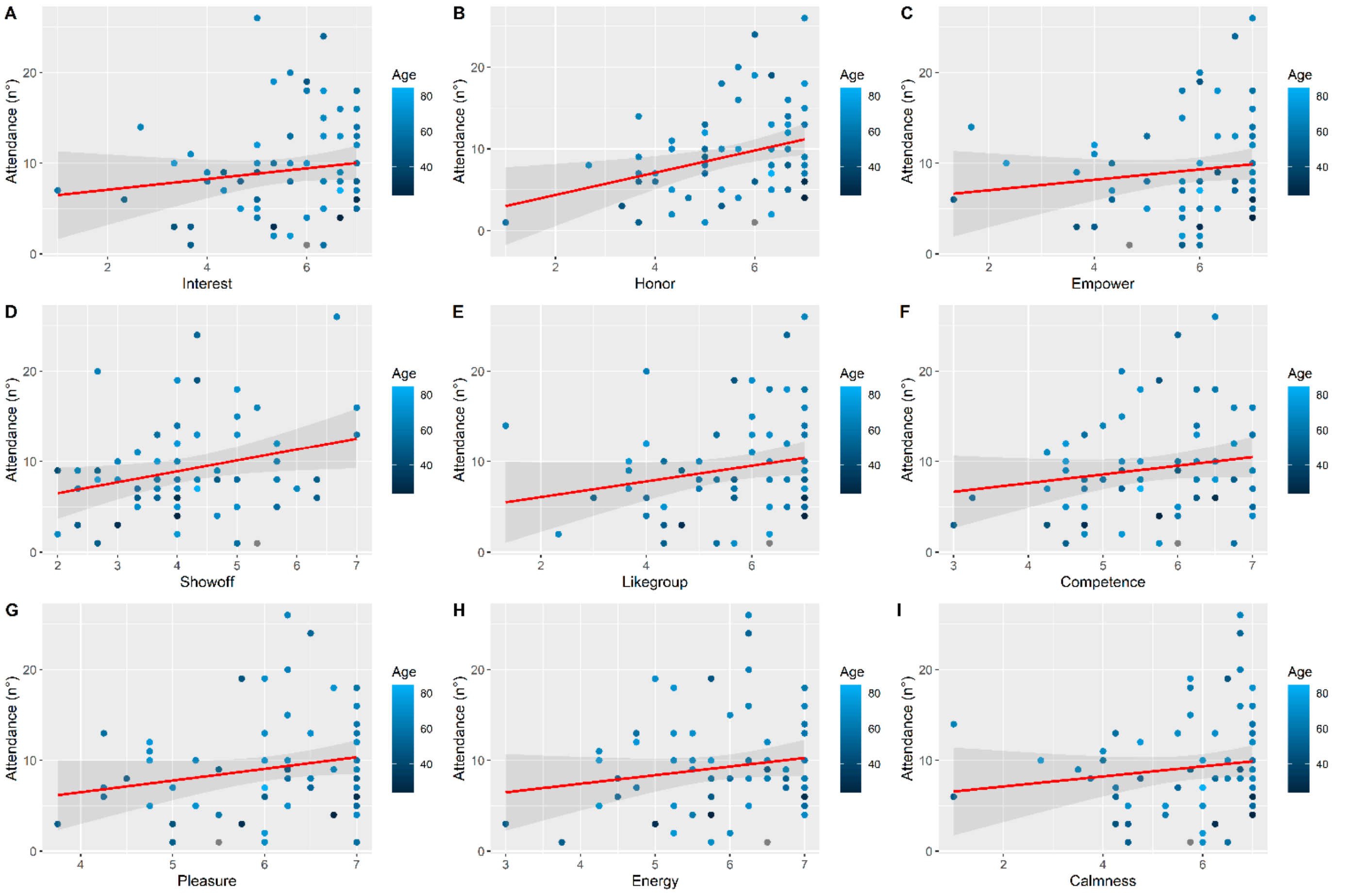

Figure S8. Relationship between attendance, weekday and temperature

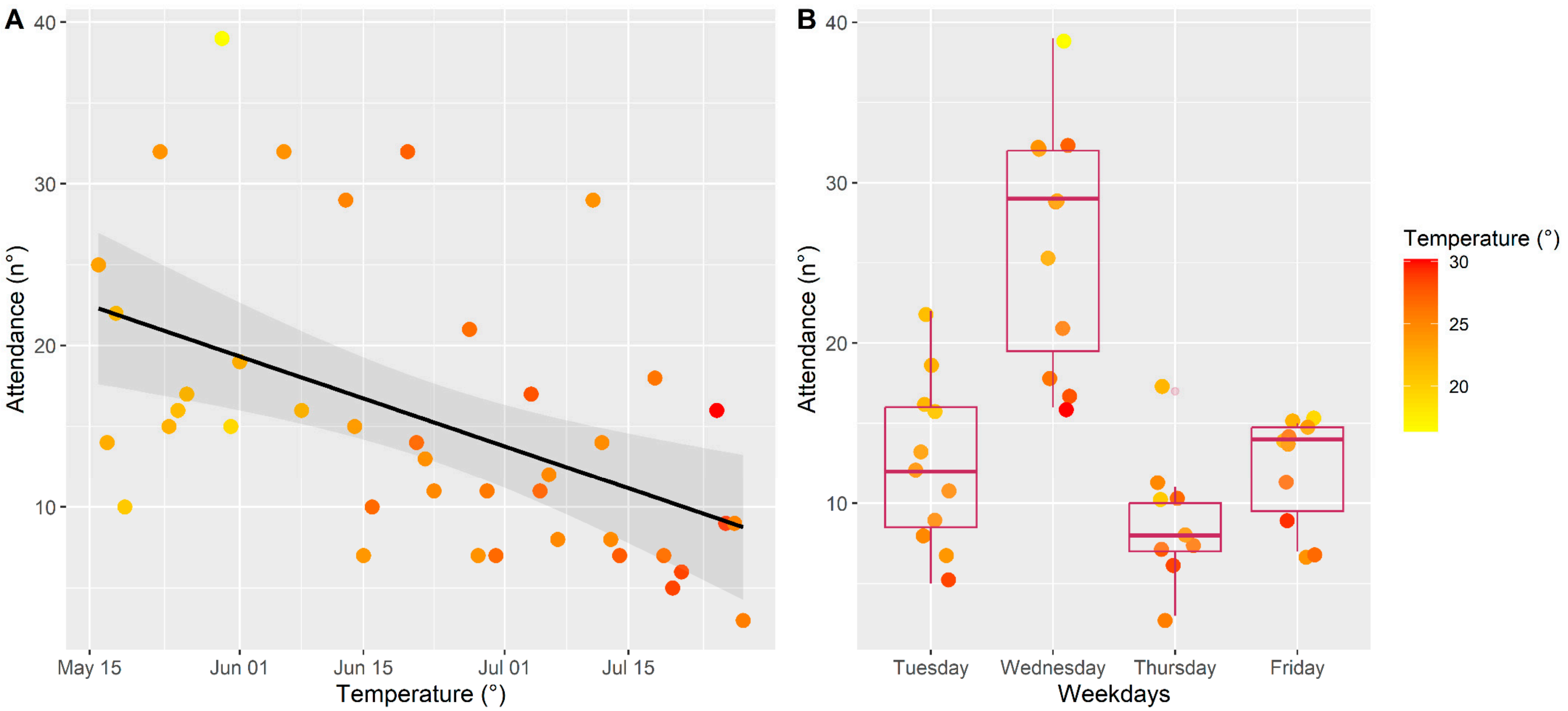

Figure S9. Percentage of session attendance per weekday

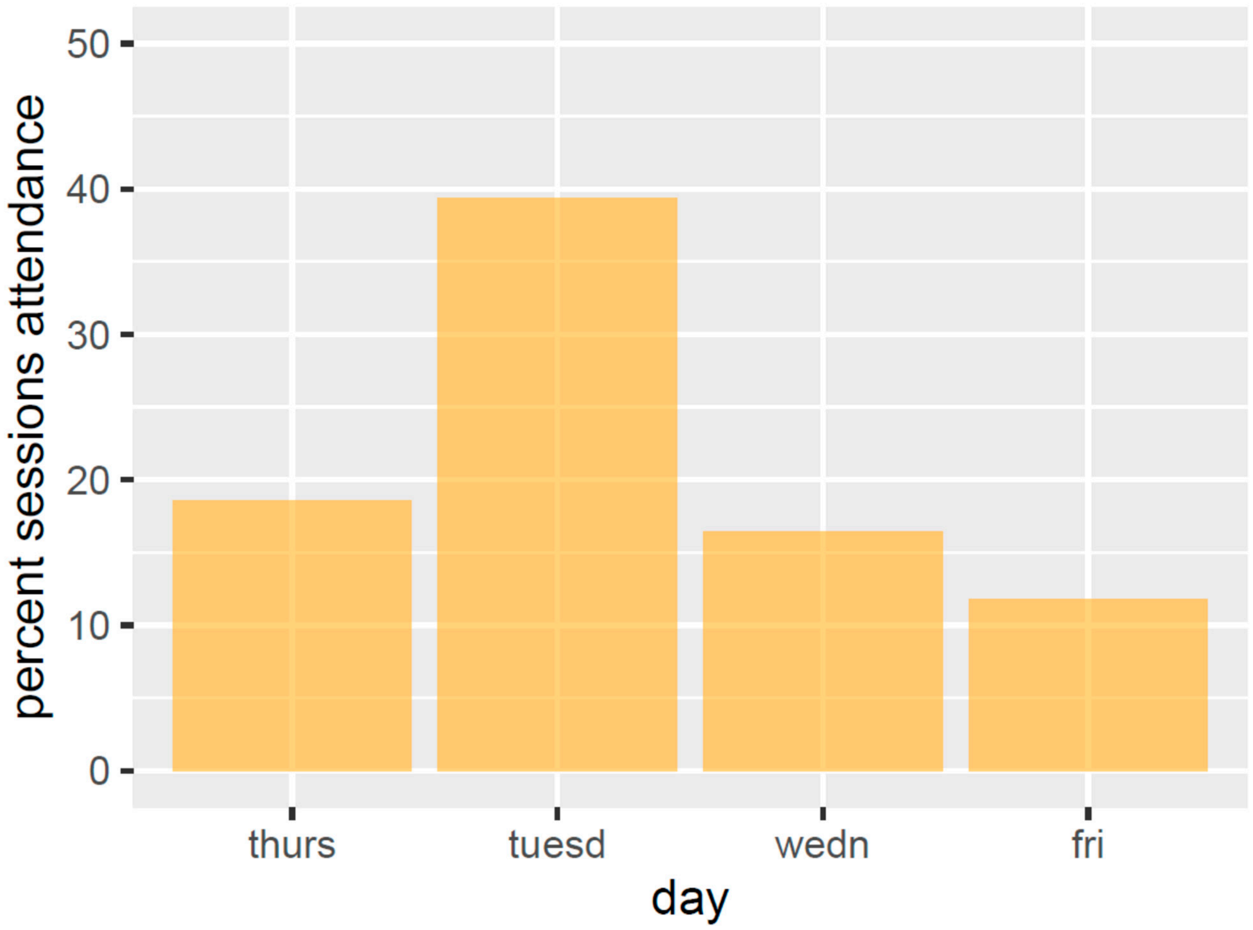

Supplement: Supplementary file 1 [file ejihpe-15-00031-s001.zip › ejihpe-3401031-supplementary.pdf]
